# Framing health and foreign policy: lessons for global health diplomacy

**DOI:** 10.1186/1744-8603-6-14

**Published:** 2010-08-22

**Authors:** Ronald Labonté, Michelle L Gagnon

**Affiliations:** 1 Department of Epidemiology and Community Medicine, Canada Research Chair, Globalization and Health Equity, Institute of Population Health, University of Ottawa, 1 Stewart Street, Ottawa, Ontario, K1N 6N5, Canada; 2 Institute of Population Health, University of Ottawa, 1 Stewart Street, Ottawa, Ontario, K1N 6N5, Canada

## Abstract

Global health financing has increased dramatically in recent years, indicative of a rise in health as a foreign policy issue. Several governments have issued specific foreign policy statements on global health and a new term, global health diplomacy, has been coined to describe the processes by which state and non-state actors engage to position health issues more prominently in foreign policy decision-making. Their ability to do so is important to advancing international cooperation in health. In this paper we review the arguments for health in foreign policy that inform global health diplomacy. These are organized into six policy frames: security, development, global public goods, trade, human rights and ethical/moral reasoning. Each of these frames has implications for how global health as a foreign policy issue is conceptualized. Differing arguments within and between these policy frames, while overlapping, can also be contradictory. This raises an important question about which arguments prevail in actual state decision-making. This question is addressed through an analysis of policy or policy-related documents and academic literature pertinent to each policy framing with some assessment of policy practice. The reference point for this analysis is the explicit goal of improving global health equity. This goal has increasing national traction within national public health discourse and decision-making and, through the Millennium Development Goals and other multilateral reports and declarations, is entering global health policy discussion. Initial findings support conventional international relations theory that most states, even when committed to health as a foreign policy goal, still make decisions primarily on the basis of the 'high politics' of national security and economic material interests. Development, human rights and ethical/moral arguments for global health assistance, the traditional 'low politics' of foreign policy, are present in discourse but do not appear to dominate practice. While political momentum for health as a foreign policy goal persists, the framing of this goal remains a contested issue. The analysis offered in this article may prove helpful to those engaged in global health diplomacy or in efforts to have global governance across a range of sectoral interests pay more attention to health equity impacts.

## Introduction

In 2007, the foreign ministers of seven countries issued the *Oslo Declaration *identifying global health as 'a pressing foreign policy issue of our time' [1]. The declaration was not the start of recent interest in health and foreign policy, but reflects a decadal trend in which health has become more prominent in global policy agendas. This prominence has been accompanied by promotion of a new concept - global health diplomacy (GHD) - to describe the processes by which government, multilateral and civil society actors attempt to position health in foreign policy negotiations and to create new forms of global health governance [2].

This article examines some of the arguments for GHD. It does not explore GHD *per se *(the 'how' of foreign policy deliberations) but several of the rationales that have been, or could be, used to position global health better within foreign policy. It seeks both to review arguments for GHD, assessing some of their strengths and weaknesses, as well as to suggest additional arguments. Its intent is to strengthen the base for those who are attempting to argue for health in a variety of foreign policy settings. Our analysis was guided by a template of major global health policy frames based on an earlier study undertaken by the lead author: security, development, global public goods, trade, human rights and ethical/moral reasoning [3]. The selection of these frames arose from the lead author's participation in international conferences and meetings on, and past research in, global health, and was refined and elaborated as part of an interdisciplinary research project on global health ethics. We make no claim that these frames are the only ones that exist; or that they are theoretically or analytically distinct. Rather, they provide useful heuristics for assessing some of what we (and others, see [4]) would contend have been the major arguments advanced for why health should be more prominent in governments' foreign policies.

## Methods

In this article we address two questions:

1. What arguments have been advanced by governments to position global health more prominently in foreign policy deliberations?

2. How does their policy framing relate to their potential to improve global health equity?

We first examined major English-language health and foreign policy statements issued from the early 2000s until 2009 (see Table 1) [1,5-13]. These statements were selected through information provided by a new World Health Organization program of work on global health diplomacy; participation in meetings and events on global health diplomacy; report bibliographies; and key word searches using Google and Google-scholar. As this was a search for government or multilateral statements on health and foreign policy academic database searches were not undertaken. Not all of these documents we reviewed carry the same political weight. Some are Cabinet-level policies or legislated requirements; others are national strategies arising from a specific sector, normative declarations, or simply commentaries by global health diplomats working within particular governments. Our intent was not to locate the forcefulness of these texts within particular government settings. Our interest was in how these documents described the different rationales for health as a foreign policy goal, and the degree to which coherence (or lack thereof) existed amongst the arguments offered. The approach was deductive using the six policy frames described earlier as a template for textual assessment. The texts were approached as interview transcripts. They were read and re-read several times, with analytical notes made concerning the arguments or rationales encountered. Key word searches of the documents using a variety of terms associated with the six frames were also undertaken, with careful reading of the text surrounding such terms in order to ensure our use of the excerpts cited in this paper are in context.

**Table 1:** Health and Foreign Policy Key Documents

Title (Abbreviated)	Country, Year	Comment, Source
Swiss Health Foreign Policy: Agreement on Health Foreign Policy Objectives * [5] (FDHA)	Switzerland, 2006	Published by Federal Office of Public Health and Federal Department of Foreign Affairs

Health is Global: a UK Government Strategy * [6,7] (UKHG) and (UKHG Annex)	UK, 2008	Issued by the Department of Health

*Foreign and Commonwealth Office Departmental Strategic Objectives 2008/09 - 2010/11 *# [8] (UKDSO)	UK, 2008	Issued by the Foreign and Commonwealth Office

*The National Security Strategy of the United Kingdom: Security in an interdependent world *# [9] (UKFP)	UK, 2008	Issued by the Cabinet Office

*Shared Responsibility: Sweden's Policy for Global Development *# [10] (SW)	Sweden, 2003	Legislation requiring annual report to parliament on how all foreign policies worked towards goal of global development (including health)

*Oslo Ministerial Declaration--Global Health: A Pressing Foreign Policy Issue of Our Time *§ [1] (OSLO)	Norway, France, Brazil, Indonesia, Senegal, South Africa and Thailand, 2007	Statement issued by foreign ministers

*Meeting global challenges: international cooperation in the national interest*. † [11] (SW-GPG)	Sweden, 2006	Issued by the International Task Force on Global Public Goods, Swedish Ministry for Foreign Affairs

*Coherent for Development? How coherent Norwegian policies can assist development in poor countries *† [12] (PCC)	Norway, 2008	Report of a two-year all party commission, Official Norwegian Reports

*Foreign policy and global health: Six national strategies *‡ [13] (WHO-GHD)	World Health Organization	FTD draft working paper, forthcoming: Geneva: World Health Organization. Report of six countries' experiences in global health diplomacy first presented at the Prince Mahidol Awards Conference, Bangkok, Thailand, January 2009

* Official policy statement on health and foreign policy

# Official policy statement on general global development and foreign policy

§ Intergovernmental joint consensus statement

† Advisory commission reports

‡ Commentaries by government officials engaged in global health diplomacy

We also undertook a non-systematic but rigorous review of recent academic literature related to each policy framing to assess the empirical or theoretical basis for differing rationales. These rationales were then examined for their actual or potential effects on global health equity. Health equity is generally defined as an absence of systematic and remediable differences between population groups [14], that are not freely chosen and which may be considered unfair or unjust [15]. While this is only one of several goals that could have been selected, it is logically implicit in health as a foreign policy concern; and is a concept with widespread traction in national public health practice, research and scholarship. It has also been elevated to a global level in part through the Millennium Development Goals (MDGs) and the work of the recent World Health Organization Commission on Social Determinants of Health [16]. Our own use of this concept (global health equity) does not necessarily mean reductions in health inequalities, although that would be a likely effect. Instead, and following from the work of cosmopolitan theorists that emphasize the importance of "capabilities" for health rather than measurable health status itself [17-20], we refer to reductions in inequalities in the resources people need to make choices concerning their health.

### 1. Health and Security

Security, alongside development, is the most frequently encountered frame in the documents we reviewed, with the securitization of health now claimed to be 'a permanent feature of public health governance in the 21^st ^century'[21]. Although 'health security' is recent in coinage, its history dates back at least to the 14^th ^century when epidemics threatened to destabilize sovereign power and to compromise the material interests of elite groups. The response to this threat often strengthened the power of states over civil society even as it undermined citizen trust in state institutions [22], a concern that now extends to inter-state relations and who gains most through collaborative efforts to control pandemics [23]. The principle contemporary arguments pertain to *national and economic security *(key arguments or rationales within each policy framing are *italicized*), echoing the historic concern over the role disease might play in economic decline and regional conflict (UKHG, OSLO):

A healthy population is fundamental to prosperity, security and stability ... In contrast, poor health does more than damage the economic and political viability of any one country - *it is a threat to the economic and political interests of all countries *(UKHG, p. 7 emphasis added).

Empirically, evidence of the link between conflict and disease remains robust [22] although the reverse relation is still equivocal [24,25]. Findings that disease leads to conflict are based primarily on correlations between infant/maternal mortality and the likelihood of failed states in African partial democracies; and between the prevalence of HIV/AIDS and civil conflict [26,27]. The latter finding corroborates historical evidence that it is the novelty and lethality of pathogens that disrupt societies and threaten political power, rather than disease prevalence *per se*. The existing wealth and stability of state institutions can moderate these effects [22], and not all analysts are convinced that the link between HIV/AIDS and state instability is as strong as has been argued [23]. At the same time, unchecked contagion within borders has been argued to engender social 'chaos' leading to increased identity-based (ethnic/class) conflicts while decreasing productivity and prosperity upon which social harmony is in part based [22]. Thus, while contested, health security concerns with disease and conflict are not unfounded.

The rationale for intervening in epidemics in foreign states follows three main logics. First, epidemic-associated national conflicts could become regional. Contemporary evidence of epidemics leading to inter-state (as distinct from intra-state) conflict is weak [22]; however, disease-amplified shifts in regional balances of power could affect foreign economic interests. Second, epidemic-associated poverty could abet a growth in terrorist activities and thus threaten national security. Poverty, either as a cause or an effect of epidemic disease, is not associated with terrorism *per se*, but impoverished regions of poorer countries have been argued to afford sympathetic (or coerced) havens for terrorist groups (UKFP), for which there is some empirical evidence [28]. Third, epidemic-associated national or regional conflicts can create peace-keeping costs to other countries, threaten citizens and military abroad and (even without conflicts) dampen economic growth and increase poverty, reducing potential markets for other countries' exports (threats to economic security), all points argued by the US National Intelligence Council in 2000 [29] and reaffirmed in the 2010 Obama Administration's National Security Strategy [30].

Three other security rationales were offered in the documents we reviewed. The first, *conflict prevention*, regards health as a means to prevent recurring conflict when rebuilding failed states or reconstructing after disasters (FDHA, UKHG, OSLO). This argument is similar to the older concept of "health as a bridge for peace," which emphasized the role of health interventions (such as vaccinations or humanitarian emergency care) as a way of reducing conflict and promoting peace; however, evidence for sustained peace resulting from health interventions is weak [23]. At issue remains the extent to which health interventions, during or post-conflict, are designed to promote the conditions for peace or are primarily a means to gain the support of non-combatant populations caught in the middle of conflicts [22]. *International humanitarian law *provides a second argument (UKHG, PCC). It lays out the rules for the conduct of hostilities and, with it, obligations on states for certain forms of protection to non-combatants. Reference here is made to the 2008 *Convention on Cluster Munitions*, in which Norway is claimed to have played a prominent role [31]. The UK policy commits to the ratification of this Convention and further calls for a legally binding treaty for the international trade in conventional arms without impinging upon 'legitimate, responsible defence exports' (UKHG, p. 21). This global health goal is reiterated in the UK's overall foreign policy initiative (UKDSO), but what remains problematic is the meaning of 'legitimate, responsible defence exports.' The UK is one of the world's largest arms exporters and has come under criticism for failing to enforce many of its own policies including those dealing with corruption or export to countries where there is risk of arms use to repress human rights [32]. France, another GHD-espousing country, similarly scores poorly for the scale of its arms exports to countries with poor democratic accountability [33].

The last of the security arguments, *fear of disease pandemics*, recurs most frequently in the documents we reviewed (FDHA, OSLO, UKHG, WHO-GHD). Epidemic-induced fear has vigorous historic precedence, and is credited with contributing to the chaos and unravelling social contracts between states and their citizenry that characterized early 19^th ^century Europe [22]. SARS and persisting concern over pandemic influenza are the contemporary flashpoints. Thailand and the UK both credit SARS with initiating their efforts in global health policy, and their adoption of the (revised) International Health Regulations. Efforts against such threats or risks are described as 'national health security,' a variation of a government's overall obligation to defend 'the state from external attack' (OSLO). But national health security is no longer a matter of one state or government alone; it has become inherently global, the common argument being that 'global health security is only as strong as its weakest link' which must be strengthened through 'global mechanisms and other measures that enable countries to make an informed and coordinated response' (OSLO). Global health security is evocative of the older concept of collective security, which describes international (and often legal) agreements amongst states to protect themselves against the actions of other states [34,35]. The UN system, notably through its Security Council, is emblematic of collective security insofar as the security of member states is presumed to require a high and somewhat binding level of international cooperation. Global health security pitches itself in a similar fashion, insofar as it emphasizes the interdependency of health risks across nations. Global health security, however, cannot yet be considered truly collective given the small number of nations that have so far committed to it; the concept of a 'concert' of like-minded nations fits better.

#### Global Health Equity Concerns

International relations theory generally ranks foreign policy goals in a hierarchy of descending importance from national and economic security (material interests/high politics) to development concerns and human dignity/humanitarian aid (normative values/low politics). The assumption is that high politics framing is more likely to lead diplomacy and policy decision-making than low politics framing [36]. But what happens when the high politics of national security and economic interests collide with the low politics of global development and humanitarian aid? It may be possible to argue national security interests for most health aid, at least over the long-term [22], but this risks rendering the concept of national security imprecise if not meaningless [24]. Since narrowly-construed domestic interests already trump those of longer-term global health need [37], aligning global health with high politics could triage assistance even further away from need. As one indication: the securitization of health disproportionately directs funding and attention to those ills deemed politically to be national security risks. Funding for HIV/AIDS (twice cited by the UN Security Council as a threat to security) and for pandemic influenza (relative to global burden of disease) are the present exemplars; they are also the only two issues to which France has attached 'thematic ambassadors' working between its Ministries of Health and of European and Foreign Affairs (WHO-GHD). Historically, national self-interest (security) has failed to motivate sustained commitment to international health cooperation [24], a point noted by some policy statements (e.g. OSLO). The securitization of health also pushes responses away from an ethos of altruism to one of self-interest, and from civil society to intelligence organizations, potentially triaging intervention on the basis of individuals' rank within military, political or economic hierarchies [38]. Its focus on infectious disease reflects more the interests of wealthier countries (with a present low burden) than of poorer countries with existing high burdens; at least to the extent that interventions are based more on outbreak containment than outbreak prevention [39]. While the newer concept of global health security could confront these limitations, its embrace of a 'weakest link' argument still privileges risks to others and not to those who may be the cauldrons of that risk. Curiously, little mention can be found in policy statements of *human security*. In contrast to national security, human security focuses on the protection of 'the vital core of all human lives in ways that enhance human freedoms and human fulfilment' [40,41]. Human security is people- rather than state-centred, with emphasis on vulnerable populations. While no longer as fashionable in foreign policy circles as it was in the late 1990s, positioning security in human terms places foreign policy consideration into a larger set of international responsibilities, creating an argumentative path into other global health policy frames.

### 2. Health and Development

The most prominent of these other frames is development. Health has long been one of the desired outcomes of development with recent studies affirming that state *investments in health *and education have been important in explaining why some countries have experienced rapid economic growth, while others have not [42,43]. These findings reverse conventional wisdom: health is no longer simply a consequence of growth, but one of its engines. This argument is posited as one of the major reasons for advancing health in foreign policy (OSLO, UKHG). As Norway's foreign minister noted in tacit acknowledgement of where global power lies (markets, and those who dominate them):

We need to find new ways of portraying health expenditures as more than costs, but also as an investment. [W]e need to. .... get to the core of the economic dimension and speak a language that people with power really understand [44].

Based on the documents we reviewed, two rationales for health as development dominate: *aid for economic return *and *aid for strategic (security, resource) purposes*. Both rationales would see development investments allocated by donor self-interest which may (or may not) reflect global health need. The investment argument for global health development (traditionally a low politics concern) overlaps with the high politics arguments of national security. As the UK policy comments, 'improving global health is vital if we are to achieve the Government's domestic and international objectives,' which hints at national security issues (UKHG). More explicitly, the UK policy is expected to cohere with that country's 'first' *National Security Strategy*, the opening statement of which - is clear: 'Providing security for the nation and for its citizens remains the most important responsibility of government' (UKFP, p. 3). Pandemics are lumped together with 'international terrorism, weapons of mass destruction, conflicts and failed states...and trans-national crime' as the modern threats to security, actions on which are justified in relation to the 'most important responsibility of government' -protection of British citizens. This justification may explain why non-communicable diseases rank low in aid and development discourse, and are completely absent from the MDGs. Chronic diseases pose less risk to national or global (trans-border) health security than do infectious pandemics. This creates incoherence within UK policy: to promote health equity, which is normative and free of condition (UKHG), and the constrained logic of security with its first priority to what will protect British citizens (UKFP).

Yet there is also *normative and ethical reasoning *underpinning (at least some) development intentions and investments. Norway has highlighted the importance of assistance to countries to reach MDG 4 (reduce child mortality by two-thirds) and MDG 5 (reduce maternal mortality by three-quarters) (WHO-GHD), targets unlikely in the short term to benefit high-income countries either in terms of new markets or reduced national (pandemic) security risk. The *Oslo Declaration *similarly was specific that donors must 'push development cooperation models that match domestic commitment and *reflect the requirements of those in need and not one that is characterised by charity and donors' national interests*' (OSLO, p. 1373-1378 emphasis added). It remains moot the extent to which such statements give rise to actual aid policy change.

The *Oslo Declaration *states the need to 'honour existing financial commitments' and it is here that actions for many countries have lagged well behind proclaimed intent (and this before the global financial crisis began to threaten future aid disbursements). Neither is it clear whether a country's official policy commitment to global health necessarily equates to an increased volume of health aid. The Swiss government policy emphasizes improving 'the *efficiency *of multilateral players in the fields of health, development cooperation and humanitarian aid,' but not aid volumes noting that 'no additional human or financial resources are planned for the implementation of this agreement' (FDHA, emphasis added). This undermines at least one component of its policy's stated objective, notably 'to strengthen the global partnership for development, security and human rights, making a credible and acknowledged contribution' (FDHA). Its major development contribution is cited as support to the Global Fund (WHO-GHD), but this support compares poorly to other countries claiming alignment with the 'health is global' concept [45,46].

The International Health Partnership+ (IHP+), as one example of a development approach to global health policy anticipated by the *Oslo Declaration*, similarly remains equivocal over whether it will deliver more health aid or only improve the efficiency and effectiveness of what is currently on offer. Launched in September 2007, with leadership from the UK and Norway, the IHP+ intends to operationalize the *Paris Declaration on Aid Effectiveness *within the health sector. The *Paris Declaration *emphasizes the 'harmonization' of activities by donors and external agencies, a response to the growth in bilateral health aid and independent global health initiatives that is weakening recipient countries' capacities to develop their own comprehensive health system plans. Harmonization, as the UK policy explains, should lead to 'international development agencies pooling a greater proportion of their money to finance directly the budgets of health sector plans in developing countries' (UKHG). Alongside harmonization is 'country-ownership' of health plans, the 'alignment' of external assistance to country priorities, and sustained and predictable donor funding. While still in its infancy, the IHP+'s first Ministerial Review in February 2009 emphasized aid effectiveness over aid volume [47]. Its first independently managed progress report (February 2010) showed slow progress and a lack of compliance with reporting accountability by most of its bilateral donors.

While all documents reviewed stressed the importance of aid, some were critical of its overemphasis reflecting renewed critiques of aid-dependency and failure (at least in the case of the African continent) to lead to sustained economic growth and development [48]. As Norway's Policy Coherence Commission reported:

The aim here is not fighting poverty through increasing aid or loans to poor people or countries, but framework conditions that can make it easier for these countries to create long-term economic growth and reduce poverty themselves.... Aid can be a crucial and necessary catalyst for contributing to development, but it is far from adequate as a tool to make this sustainable (PCC, p23).

As one of several instances of these 'framework conditions' the Commission assessed Norway's foreign direct investment strategy. It found that very little of Norway's foreign investment goes to Africa and much of what does is in oil production, which so far has failed to develop African economies. Even so, the small amount of such investment is greater than the (comparatively generous) amount of aid that Norway provides to Africa, 'which illustrates how marginal the scope of the aid is in relation to other resource flows to developing countries' (PCC, p. 27). The Commission recommended that Norway's large 'Government Pension Fund - Global' be used more strategically for investments that benefit primarily the poor; that a large fund be created for investments in Africa and least developed countries; and that emphasis in both should be on environmentally sustainable forms of economic growth and development. These recommendations were further qualified by reference to foreign direct investment yielding its greatest development potential through transfer of new technologies and managerial skills; improved social, environmental, gender equality and labour standards; provision of decent employment; inter-linkages with the local economy; and payment of taxes and royalties that contribute to domestic development financing. There were dissenting opinions to these recommendations amongst Commission members; and the Commission, while all-party, was advisory only and does not reflect Norwegian foreign policy. Nonetheless, these recommendations show the potential breadth of engagement in policy coherence for development in which improved health equity is considered an integral component.

#### Global Health Equity Concerns

If one accepts donor governments' endorsement of the MDGs and the 'weakest link' global health security argument, aid in general and health aid in particular should be allocated by global health need. The 2007 OECD-DAC Report did find that 'the "poverty-efficiency" of ODA,' the amount disbursed by poverty need, 'is continuing to increase' [[45], p.20], poverty being the major risk condition for high disease burdens. The baseline for ODA poverty-efficiency, however, is very low; and current development practice, while improving for health more than for other sectors, remains driven by foreign policy objectives largely removed from demonstrable need [23]. Efforts to bypass the partisanship of bilateral aid have seen a recent and dramatic rise of disease-specific global public-private partnerships in health, now numbering over ninety [49]. This growth has been defended on the basis that 'fighting against diseases (especially contagious diseases) is a global public good' (our next policy frame) and the existence of 'reasonable doubts about the levels of efficiency and effectiveness of traditional aid channels' [[50], p.11]. At the same time, this proliferation in such initiatives compounds the fragmentation problem and increasing transaction costs of health development assistance. Issues in global financing for health are long-standing and well-argued elsewhere [51]. We will not enter these debates in this article apart from noting three key points. First, recent reviews suggest that health aid has played an important part in improving outcomes in many recipient countries, particularly when it is additional to increased domestic spending on health [52]. It also slows the out-migration of health workers in severely under-resourced nations by creating conditions more favourable to their retention [53]. Second, the argument that Africa's inability to develop despite receiving approximately USD 1 trillion in aid transfers over the past 40 years, the basis of most critiques of aid ineffectiveness, is undercut by studies finding that almost double that amount in capital flight left the continent over the same period [54]. Much of this financial impoverishment was the result of multinational tax avoidance aided by the persistence of offshore financial centres based in, or under the protectorate of, high-income donor countries. This is one indication of foreign policy incoherence on a grand scale. Third, development financing has become increasingly framed by reference to performance-, results- or outcome-based criteria. The argument for results is in line with the GHD concern that aid must be shown to 'work' in order to 'retain the support of taxpayers' (WHO-GHD). If genuinely involving 'country-ownership' in criteria definition [55] such measures can allow for a better assessment of aid effectiveness and avoid problems of fungibility, where donor funding allows diversion of public revenues into other forms of spending of less developmental value. Carried to an extreme, however, results-based requirements would favour projects with short-term deliverables at the expense of long-term infrastructure, or those countries with greater existing capacities to show returns at the expense of more vulnerable states.

### 3. Health and Global Public Goods

The concept of global public goods (GPG) offers one of the potentially strongest arguments for GHD. A public good has two features: Its use is open to all, and does not diminish through use by others [3]. There is no consensus on the boundaries demarcating a 'global' public good or its corollary, a global public bad; but by narrow economic definition 'there are only a few "pure" global public goods...peace and security, protection against and prevention of the spread of epidemics, financial stability and fundamental human rights, a stable climate, free access to knowledge, opportunities to travel freely and globally agreed rules on trade and investment, all have characteristics of such goods' (PCC p. 23). Public goods classically arise from market failures due to free-riding, where those not paying for the good nonetheless benefit from its presence thereby leading to its undersupply; and from externalities arising from market transactions that create a public bad, such as pollution. These failures are only overcome by public provision or regulation as a form of collectivization of both costs and benefits.

The term 'global public good' was infrequently cited in the documents we reviewed, the exceptions being the PCC and the SW-GPG, both of which were not official government policy statements. However, frequent reference to a number of GPGs was made in all of the documents suggesting implicit acceptance of the concept. The one most cited was *prevention of pandemics*, with the role of the International Health Regulations (IHR), and its reporting obligations on nations, as an exemplary global public good (FDHA, OSLO, UKHG, WHO-GHD); although the Swiss policy justifies its IHR ratification by reference to the need to protect 'the health interests of the Swiss population' (FDHA p.14) rather than to encourage a greater supply of GPGs. In a more multilateral vein, the UK policy emphasizes the importance of the IHRs as providing 'the essential framework within which the world can better manage its collective defences against acute public health risks that can spread internationally and devastate human health, while avoiding unnecessary interference with international traffic and trade' (UKHG Annex p.24). The reference to trade has historical meaning; the first International Sanitary Conference in 1851 took place against a backdrop of the increased global movement of goods leading to greater risk of disease pandemics such as cholera, plague and yellow fever. The merchant class was sceptical of state quarantine measures, especially if applied differentially by countries, and pressed for international cooperation to prevent such risks in a way that would not affect global trade [56,57]. Where the new IHRs differ from former reporting requirements is in a change in diseases for mandatory notification and a more generic requirement that countries report any 'extraordinary public health event which constitutes a public health risk to other States through the international spread of disease, and may require a coordinated international response' [58]. While there is no enforcement measure for the IHRs, the ability to use non-governmental sources of information and the inherent reciprocal self-interest is presumed to offer sufficient incentive for compliance. This may overcome free-riding, but it does not address the 'weakest link' problem associated with GPGs, in this instance the lack of resources for pandemic preparedness in many of the countries that are most likely to be sources of new pandemics.

That weakened national public health goods can erode GPGs leads to the argument that *provision of assistance to prevent such epidemics *through strengthened public health systems in low- and middle-income countries is an essential requirement (SW-GPG). Yet most health aid presently goes to particular disease programs or to health *care *strengthening; very little goes to public health interventions that create national public goods (e.g. sanitation, potable water, slum upgrading, disease surveillance and monitoring, public health regulations). It was the strengthening of such measures that reduced communicable disease and improved life expectancy in industrializing countries in the 19^th ^century, and that is doing the same in those developing countries today that are attempting to follow a similar path. There is also evidence that such national public good/public health programs are relatively inexpensive, while the economic savings resulting from the prevention of disease are substantial [59].

A *stable climate *is another GPG, the importance of which is cited in several documents (SW-GPG, OSLO, PCC, UKHG, WHO-GHD). The UK strategy gives considerable attention to climate change and mitigation strategies to prevent conflict over natural resources, and emphasizes using evidence of the health impacts as a means of motivating more international action on reduction and mitigation (UKHG). Other statements (SW, SW-GPG, PCC) generally acknowledge the need to advance mitigation and adaptation efforts and for resource transfers from richer to poorer countries to assist this. Yet evidence of action is less prominent, partly attributed to richer countries being less affected by climate change in the short-term, or sufficiently so for it to become the high politics of national security (PCC). As of 2009, less than 10 percent of donor pledges to developing countries to cope with climate change were disbursed [60]. Neither is it clear if the recent proliferation of climate change and environmental funds will be at the expense of other forms of development assistance, rather than represent new funding [61]. Where there is less doubt is the inadequate scale of the pledges, even assuming they are all kept, leading to 'calls to scale-up current finance levels by two orders of magnitude, from hundreds of millions to tens of billions a year' [62].

*Regulating health-damaging products *also fits within the definition of a GPG. The adoption of the Framework Convention on Tobacco Control (FCTC) in 2003 is regarded as one of the most important ventures into global health regulation by the WHO and one of the key moments in GHD. The FCTC, however, avoids any reference to trade, despite strong evidence that trade in tobacco increases smoking rates [63]. In effect, the most important global dimension of the tobacco problem disappears in a series of requirements for domestic regulation. While the World Trade Organization has stated its deferral to the FCTC if a tobacco trade-dispute should arise amongst members, there remains concern that provisions in the Agreement on Trade-Related Intellectual Property Rights could be used by tobacco firms to challenge domestic requirements for warning labels on cigarette packages. Bilateral investment treaties, which permit corporations to directly sue national governments over alleged treaty violations, pose a more serious challenge. In early 2010, the tobacco multinational, Philip Morris, launched a suit against the government of Uruguay over its aggressive warning label requirements, claiming it infringed the intellectual property right of their trademark logos protected under a bilateral investment treaty between Uruguay and Switzerland [64,65]. Another limitation of the FCTC is that it lacks enforcement measures for countries that fail to abide by its protocols. The potential force of the convention's reporting requirements and their use by civil society organizations (CSOs) have nonetheless engendered calls for similar conventions on alcohol and its global trade [66,67] and on the globalization of food commodity chains creating obesogenic environments [68].

#### Global Health Equity Concerns

A major equity concern with GPGs is that the governance frameworks for such goods, such as the IHRs and the FCTC, are potentially weakened by their 'soft' law status. To some engaged in GHD, this 'soft' law is an advantage, providing greater flexibility for advancing health concerns in foreign policy negotiations without having to continually check with political decision-makers over what might become binding treaties: '[I]ncreased use of legal solutions that are not binding, such as "codes," as opposed to formal agreements, will allow progress to be made more rapidly, and with greater emphasis on consensus than would be the case if conventional treaties were prepared'(WHO-GHD).

The potential conflict between such codes and the 'hard' law of trade treaties (the next policy frame we consider) questions such an assessment. An example of hard law/soft law conflict exists in the issue of transparent information sharing (essential to the IHRs), intellectual property rights and the power differentials between high-income and low-/middle-income countries. While not formally part of the IHRs, countries worldwide have been collaborating with the WHO in sharing viral samples as part of a process to prepare for a future pandemic influenza. In 2007 Indonesia, a potential epicentre of any future pandemic, stopped sharing viral samples with the WHO because they were being used by laboratories to create patented drugs the country could not afford to purchase. WHO agreed to revise the terms of reference for collaborating laboratories to which such samples were sent. But WHO-hosted intergovernmental negotiations have so far failed to reconcile developing country interests in benefits-sharing with developed country demands to retain intellectual property rights over eventual vaccine discoveries [69], an instance where private economic interests (economic security) and its 'hard' law trade treaty protection will almost certainly impede the provision of GPGs and their 'soft' law codes of practice. Even the emergence of pandemic H1N1 (when concerns over its virulence were still high) failed to break this deadlock [70]. Thailand has been particularly critical on this account:

Many developing countries... have proposed that companies or research institutions should not be allowed to lay intellectual property claims on products derived from shared biological specimens...It will take a lot of work and diplomacy to show that it makes more sense to defend public goods instead of private interests... but the costs in human terms associated with collective health insecurity clearly outweigh any gains or considerations in protecting intellectual property (WHO-GHD).

Perhaps because it was advisory to government in a policy decision-making role, the Norwegian Policy Coherence Commission was straightforward on the issue of the unequal global power relations that preclude effective use or protection of global public goods in its plea for a more egalitarian approach to foreign policy coherence:

Power is systematically unevenly distributed between countries, and makes some countries dependent on framework conditions set by others. The latitude for action afforded to developing countries is, therefore, often extremely limited... Acknowledgement that conflicts of interest exist between rich and poor countries is required, *as is a willingness to consider aspects other than Norwegian interests, and to give up privileges that rich countries currently have in a number of areas*. Such changes can be painful to carry through in policy areas that apply to national interests...Nevertheless, there is no excuse for not changing a policy that thwarts development in poor countries (PCC, pp.21-22; emphasis added).

### 4. Health and Trade

Power differentials are most apparent where global health intersects with global trade. A rules-based trading system is considered to be a global public good for the decline in economic growth (a global public 'bad') that it is presumed to avoid. Generally, all policies and reports we reviewed favour an open global trading system as one that would 'support global health security' (OSLO). The UK further emphasized the need for such a trading system to be 'stronger, freer and fairer' (UKHG, p. 58). Other statements, however, were less sanguine on how 'free' or 'fair' a global trading system might be, citing continued protectionism by wealthier countries (SW-GPG) or inequalities in the power to negotiate equitable terms (PCC). Largely absent was any consideration of the role increased global trade and travel has on the risk of pandemics, despite the long history of pathogens and pestilence following trade routes and the expert concern, expressed several years before the birth of the World Trade Organization (WTO), that global trade is a major potential source of emerging infections [71]. Liberalization of food trade, and the economic incentives it creates for large scale (overcrowded) animal production and food processing, are particular worries [71].

Aside from sanitary considerations, the most important trade and health argument follows a standard economic logic: *trade liberalization increases growth and development, which reduces poverty, which leads to improved health that in turn improves growth*. The evidence base for this logic, however, is weak. While most econometric studies find that liberalization on average is associated with growth, this positive relationship 'is neither automatically guaranteed nor universally observable' [72]. Moreover, poverty reduction during globalization's peak decades of liberalized trade, during which global economic growth quadrupled, has been modest at best, leading one senior World Bank development economist to conclude that "it is hard to maintain the view that expanding external trade is...a powerful force for poverty reduction in developing countries" [73]; while there is robust empirical consensus that trade liberalization leads to inequalities in labour markets, as wages for highly skilled workers in globally competitive industries rise and those for lesser skilled workers in relative abundance fall [74]. This is not to argue that trade liberalization is necessarily bad for health; rather, there is evidence and argument that the pacing of such liberalization, alongside the provision of social safety nets and flexibilities that account for countries' different development levels and productive capacities, can help to offset the dislocations in domestic labour markets that inevitably follow openness to global competition [75,76]. These findings suggest a careful nuance of any automatic claims of liberalization's health beneficence within foreign policy considerations.

*Intellectual property rights *(IRPs) have generated the greatest health and trade controversy and the most discussion within the documents we reviewed. Arguments from high-income countries where IPRs have greater economic importance emphasize a balance between ensuring access to medicines in low- and middle-income countries and maintaining sufficient pharmaceutical profitability to stimulate new research: 'Switzerland, with its major pharmaceutical industry and long humanitarian tradition, is committed both to adequate protection of intellectual property as well as access to essential drugs for the world's poorest countries' (FDHA, p. 13), arguing that 'appropriate protection for intellectual property [is] an essential incentive for research into, and development of new drugs and vaccines' (FDHA, p. 15). The same rationale is found in the UK policy which affirms 'the right of developing countries to use the flexibilities built into the Trade-Related Intellectual Property Rights (TRIPS) Agreement, such as the judicious use of compulsory licensing' but adds that 'this should not be at the expense of damaging incentives to invest in research and development' (UKHG, p. 28). The 2001 Doha Declaration on TRIPS and Public Health to which the UK policy refers, however, makes no mention of 'judicious' use of its provisions nor the need to 'balance' use of these flexibilities with incentives to pharmaceutical company research.

*Health services are also tradable commodities *under WTO and some regional and bilateral agreements. Only the UK policy discusses health services trade, couching its economic interests as one of mutual benefits arising 'from the opportunities that come through freer and fairer global trade in health services and commodities' (UKHG, p. 9). It specifically targets the health sector in India, China and Brazil for its commercial health services and products. Yet the role of private sector involvement in health services in improving health equity remains ideologically and empirically contested, with the weight of evidence highly critical of unregulated private markets [77]. The UK commitment to increase trade in health services appears to conflict with other of its policy statements concerning the depth of medical poverty created by private health care; and commitments to strengthen through its development assistance public health systems in poorer countries.

*Poorly regulated global capital flows *pose substantial health risks, likely much greater than liberalized trade in goods [78-80]. Portfolio investment (essentially trade in currencies) dwarfs all other forms of capital flows. Such speculative capital flows are subject to panics, manias and crashes [81] with devastating effects on health through depreciation of national currencies and purchasing power [82,83], the most recent (and still ongoing) global financial crisis being a case in point. Subsequent austerity measures reduce public revenues or expenditures on health and social program transfers [84-86]. The UK policy is alone in referencing 'global financial turbulence', for which it calls for non-specific reforms of the IMF (UKHG Annex, p.49). Given that it is the most recently released statement on global health policy that we reviewed, the silence on this issue attests to the general lack of national regulatory oversight of financial markets until their rapid collapse in 2008.

#### Global Health Equity Concerns

In terms of indirect health effects (the health externalities of increased global economic integration) trade liberalization may be associated with greater growth and poverty reduction, but the relationship is dependent on pre-existing development conditions and public policies that vary by country. Increases in economic insecurity and labour market losses resulting from liberalization may be offset by stronger social protection measures, but these are less affordable if developing countries are required to reduce tariffs before implementing broader and more equitable forms of capturing tax revenues [52]. While developing countries under WTO rules have been granted 'less than full reciprocation' in their tariff-reduction schedules, present negotiations for increased 'non-agricultural market access' (NAMA negotiations) could result in annual net tariff losses for developing countries of USD 63 billion, but losses of only USD 38 billion for developed countries [87,88]. The Norwegian Policy Coherence Commission was strongest in expressing concerns over the trade/health relationship. It argued that a clear conflict existed between its country's foreign policy goal to take an 'offensive interest in the NAMA negotiations' and its 'expressed policy to support developing countries' requirements and help preserve their policy space' (PCC, p.47). It further noted that a coherent trade and development policy demands 'asymmetrical agreements' disproportionately benefiting developing countries. At present, such agreements asymmetrically favour developed nations. Notwithstanding the economic gains of certain Asian and Latin American developing countries over the past decade, estimates of aggregate gains from a completed WTO Doha Development Round under the 'most realistic scenario' show developed countries by 2015 gaining USD 80 billion while developing countries would gain only USD 16 billion [55].

Countries' economic interests in trade are also in conflict with more direct pathways affecting health, notably with respect to IPRs and health services. The rationale that extended IPRs are essential to finance research and development for new drugs, especially for neglected diseases, is weak; while extended IPRs are known to reduce access to essential medicines in many countries now subject to their provision in trade treaties [89]. Similarly, growth in health services trade can bring economic benefits to certain sectors of the economy, but it can also lead to inequities in access to both providers and services by those unable to pay [66]. More at issue than health services trade itself is the extent to which international commercial exchange becomes written into binding trade treaties such as the General Agreement on Trade in Services that preclude governments from changing their minds in the future, at least in any cost-free way. This led Norway's Policy Coherence Commission to conclude that health (as well as water and education, two key social determinants of health) 'not be sectors that are subject to the GATS regulations, but be protected with a view to future generations' possibilities for regulating these basic services for the good of the population' (PCC, p. 54).

If global health equity is to be more central to the outcomes of trade, wealthier and more powerful countries need to accord greater trade policy flexibilities to poorer developing nations. The essential rationale is that 'trade is an instrument, not a goal in itself' (PCC, p. 45), a point underscored by Brazilian and Thai officials engaged in global health diplomacy (WHO-GHD). Human rights are sometimes argued as contrasting international obligations to trade treaties (WHO-GHD), yet human rights, much less specific health rights, have so far been absent in trade negotiations and missing completely in rulings on disputes [90].

### 5. Health and Human Rights

The importance of human rights was underscored in all of the documents we reviewed, including reference to *health as 'a fundamental right of every human being' *and, in line with legal scholarship, that 'life is the most fundamental of human rights, and that life and health are the most precious assets' (OSLO). France cites its support of EU policies on 'health as a fundamental human right' and gives as an example its efforts with UNAIDS to eliminate travel or entry restrictions on persons who are HIV-positive (WHO-GHD). The UK policy commits to including health as a section in its government's annual human rights report (UKHG Annex, p.2), claims to champion the rights of women with particular reference to HIV treatment and services access (UKHG Annex, p.28) and sexual/reproductive rights (UKHG, p.42), and cautions that unfair or unethical trade can deprive workers of their 'rights to security of employment and compensation' (UKHG, p.60); although how strongly this last sentiment motivates UK diplomacy in trade negotiations remains open to empirical scrutiny. Thailand claims that the *right to health *was the driving force behind its global health diplomacy efforts while Brazil finds that having the right to health in its federal constitution provides a strong base for arguing health in foreign policy agendas (WHO-GHD). The Swiss *Health Foreign Policy *states that 'one of its main objectives is to strengthen the global partnership for development, security and human rights that has been agreed upon and implemented in the context of the UN (FDHA, p.12),' although its position on IPRs is contrary to that espoused by human rights experts. Sweden's 2003 legislated *Policy for Global Development *references specific rights issues throughout, while Norway's Commission report devotes considerable attention to a human rights framing of its country's foreign policies.

Despite these frequent invocations to human rights, and apart from the Swedish and Norwegian documents, little specific reference is made to the actual international human rights framework (IFHR), its covenants and state-parties' obligations, and its reporting requirements. Central to global health in the IFHR is the right to health, technically known as the Right to the Highest Attainable Standard of Physical and Mental Health. Article 12 of the International Covenant on Economic, Social and Cultural Rights (ICESCR) obligates states to ensure equitable access to a minimum set of health services, while General Comment 14 (GC 14) identifies a broader range of actions required for the progressive realization of this right [91]. GC 14 further states that 'collective rights are critical in the field of health' [91] implying a need to counter-balance individual entitlements, although there is no clear guidance on when an individual health right claim might compromise a collective health right claim [92].

The right to development further implies that rights are collective rather than simply individual in entitlement. Adopted by the UN in 1986, the Declaration on the Right to Development contains several articles that place stringent obligations on states parties to ensure greater equality of opportunity and equity in outcome [93]. Some legal scholars believe that this right may actually entitle poorer countries (through their state) to make claims for assistance from higher-income nations [94]. As a Declaration rather than a Treaty, the right is non-binding on states although it has become 'a focal point of United Nations human rights activity concerning development and has been reaffirmed as a universal human right by the international community [95].' Paul Hunt, former UN Special Rapporteur on the right to health, similarly argues that Article 2(1) of the ICESCR obligates 'developed States...to provide *international assistance and cooperation *to ensure the realization of economic, social and cultural rights in low-income countries,' a normative affirmation of which exists in the MDGs [96].

Rights-based arguments are not simply about health or health-care; they extend to well-being and to individual capabilities that form a base for individual and group enjoyment of health that, in turn, forms a base for the fuller enjoyment of all human rights. Chapman [97], an ethicist and human rights scholar, draws on the work of Nussbaum to argue the priority of some rights over others. Nussbaum, with Sen, developed a moral philosophy based on the concept of human capabilities. While Sen argues effectively for the obligations states have to provide a minimum basket of resources allowing people to develop their capabilities (and hence their health) [18], Nussbaum [20] attempts to identify the contents of that basket. Her list is extensive and imprecise. But, drawing from the International Covenant on Civil and Political Rights (ICCPR), as well as the ICESCR, Chapman maps these capabilities against what could be considered basic human rights for human capabilities:

1. The inherent right to life (ICCPR, art. 6.1);

2. Components of the right to the highest attainable standard of physical and mental health (ICESCR, art. 12);

3. Parts of the right to adequate education (ICESCR, art. 13);

4. The right to freedom of thought, conscience, and religion (ICCPR, art. 18);

5. The rights to peaceful assembly (ICCPR, art. 21), freedom of association with others (ICCPR, art. 22), and the right to take part in the conduct of public affairs and to vote (ICCPR, art. 25);

6. Equality before the law and the prohibition of discrimination (ICCPR, art. 26).

One could consider this a short list against which any foreign policy decision should be interrogated before being agreed upon; and which should inform global health diplomacy efforts that incorporate both health and its key social determinants.

#### Global Health Equity Concerns

One of the greatest challenges in strengthening global health as a foreign policy concern exists in the opposition between several provisions of trade treaties and obligations under human rights covenants. While there has been no shortage of efforts to position both health and human rights more strongly in trade debates, health actually 'has a much stronger profile in international trade law than the protection of human rights, which is not an objective trade treaties recognize as a legitimate reason for restricting trade' [98]. This lack of attention in trade treaties to issues of human rights has been commented upon at the highest UN levels. The former UN Special Rapporteur on the Right to Health, Paul Hunt, issued several reports noting potential conflicts between trade and health rights [99], including *a priori *advice against agreeing to TRIPS+ provisions in bilateral treaties in light of human rights obligations to ensure access to essential medicines [100]. Several UN Special Rapporteurs have detailed how trade liberalization, as generally negotiated, can undermine states' capacities for the progressive realization of a number of human rights [97].

While the relationship of human rights covenants to other treaties, such as trade agreements, is still a central debate in international law, the primacy of human rights is supported by legal and scholarly texts. Section 103 of the *Charter of the United Nations*, for example, states that in conflicts between Charter obligations and those under other international treaties, Charter obligations will prevail; while the *Vienna Declaration and Program of Action (19*93) is widely regarded as a state consensus on the moral primacy of human rights over other public interests. One hundred and seventy-one states proclaimed the *protection and promotion of human rights *and fundamental freedoms as the first responsibility of governments [101].

The *Vienna Declaration *sits uncomfortably with the high politics of national security and economic interests. It is possible to shoe-horn both security and economics into several human rights treaty obligations; but it has also been argued that international law 'presupposes that there is a minimum substantive normativity inherent in the international legal order, a kind of foundation or floor, grounding the aspirations and efforts of the international legal system', and that the preservation of human life and health can be understand to comprise that floor [[102], p.10]. Human security, or the security of the person, is not the same as national security. It implies a different set of policy priorities and diplomacy approaches than arguments from national security alone.

The principal health equity concern with the rights frame is that the right to health can be interpreted in legal decisions as an individual right only. This could configure health services in response to litigation that may give rise to inequities in access for others. Evidence of this is found in countries that have made this right justiciable within their constitutions, such as Brazil, where this right has become 'a strategy of the pharmaceutical industry, to take advantage of the large number of judicial decisions granting individuals a right to receive expensive medicines this industry produces' [103]. The cost to the public health systems of financing such patent medicines may be at the expense of expansion of primary health care services known to disproportionately benefit the poor. International legal scholars argue that human rights emphasis should be placed on poorer and more vulnerable populations. This requires greater attention to the concept of collective rights, implied in GC 14 on the right to health and explicit in the Declaration on the Right to Development. Finally, despite evidence of rights-based arguments having legal weight within countries, and the exercise of such rights associated with better population health outcomes across a range of countries [104], there is no formal mechanism for their enforcement outside of national jurisprudence. Globally, they remain 'soft' law, albeit representing 'the most globalized political value of our times' [105].

### 6. Health and Ethical/Moral Reasoning

The legalistic language and problematic individual nature of human rights has some scholars claiming that without *a more explicit set of ethical principles *against which decisions can be appraised, the high politics of foreign policy might always override the low politics of global health. Arguments from values or ethics are rare in policy discourse [106]. Values may be perceived as vague or not universal (cultural relativism), a feature of particularist moral philosophy [107]. However, states, the people who govern them and the institutions they create are moral actors not exempt from a capacity for, and necessity of, ethical justification for their actions.

Explicit reference to values or ethical norms was not common in the statements we reviewed, but neither was it absent. The UK *Security Strategy *claims that it 'is clearly grounded in a set of core values' that 'include human rights, the rule of law, legitimate and accountable government, justice, freedom, tolerance, and opportunity for all' (UKFP, p. 6) - an ill-defined shopping list. Sweden's 2003 policy more restrictively describes 'the firm conviction that everybody has a right to a life in dignity' as 'the basis of the solidarity with poor, oppressed and vulnerable people' (SW, p. 19).

Health has special importance to an individual's experience of security or dignity [19]. The reasoning for this lies, first, in health as basic to peoples' enjoyment of other rights or capabilities; and second, in provision of resources for health being prone to market failures requiring collective forms of intervention. These instrumental arguments are accompanied by an 'ethical principle of human flourishing or human capability' with roots in Aristotelian political theory and Sen's and Nussbaum's capabilities approach [18,20]. Health is both 'intrinsically and instrumentally valuable' [[108], p. 430]. But achieving it demands resources for capabilities. This immediately surfaces questions of *social justice *in how fairly (equitably) resources are allocated amongst peoples and, from a global health equity perspective, countries.

Social justice is argued to be a universal concern, since all social arrangements to be legitimate and to function must attend to issues of equity [17], although there are subtleties in how social justice theory is conceived. Two main dimensions exist: equality of opportunity, achieved through procedural justice that treats equals the same; and equality of outcome, achieved through substantive justice that treats unequals differently according to their initial endowments or privileges. Both equalities (opportunity, outcome) are ideal types; neither exists in 'true' form. They represent aspirational ideals of what societies strive to create for their members (fairness in outcomes) and how they believe this should be accomplished (fairness in opportunity).

*Moral arguments *underpinning a superordinance of procedural over substantive justice have strong roots in Western theories of liberal individualism, but do not entirely discount the importance of redistribution. Smith, in his *Wealth of Nations*, famously argued for some form of state intervention to moderate the market's 'invisible hand' [109].

More recently, Singer, in a utilitarian vein, posited that it is both just and of collective benefit to act to relieve poverty and deprivation if, in doing so, we do not sacrifice something of comparable moral significance [110]. Rawls in his influential *Theory of Justice *argued that people, standing behind a 'veil of ignorance' as to their social standing at birth, would choose a justice that guaranteed a minimum of primary goods basic to their needs; and that inequalities in such goods are allowable only to the extent that they improve the lot of the least advantaged compared to what their lives would have been like without such inequalities. This 'difference principle' obliges minimal interventions of redistribution and regulation, as well as procedural justice [111]. Rawls' indifference to matters of escalating inequalities that could undermine human agency (and hence health) is one source of criticism of his moral philosophy, as was his initial exclusion of health care (and by extension other resources for health) as primary goods.

Rawls' justice theory is located within the 'social contract' school, which views states as the primary actors in international relations, consistent with dominant international relations theories and the classic hierarchy of foreign policy goals. It elides with a particularlist justice perspective in which communities (or nation-states) with shared meanings and practices set the political boundaries for moral arguments: there are no universal moral principles of justice; only those relative to particular peoples and places [107]. Pogge [112] challenges this by drawing on cosmopolitan arguments and the existence of human rights treaties. Cosmopolitanists (which include Singer, Sen and Nussbaum) hold that 'the ultimate units of moral concern are individual people, not states or other particular forms of human association' [[113], p.470], a position similar to that of human security. Held [113], another cosmopolitanist, further argues that the existence of national or local decision-making having trans-local effects demands that 'political institutions need not only be locally based but must have a wider scope and framework' in order to respect the inclusion and agency of 'people who are significantly affected by a political issue in the...transcommunity public...sphere' [[113], p.471]. Pogge weds this argument to a claim that human rights constitute a universal moral standard for all individuals. In doing so, he extends Rawls' basic justice theory to a global level, contending that there are not simply 'positive duties' to assist (setting aside debates as to the level to which such assistance should rise), but moral obligations (negative duties) to prevent harm. Pogge's theory of relational justice is based on three lines of argument:

1. The radical inequalities observed between peoples and nations today are partly an effect of a violent history in which some gained at the expense of others. While we individually cannot be held responsible for the actions of our forebears in this 'conquest,' as moral persons we can be held accountable for rectifying the vast disparities in initial conditions that this history has created - a point at least nominally ceded to by high-income countries accepting greater responsibility for climate change mitigation based on their past excesses in emissions and economic benefits derived from them.

2. Not only does procedural justice by itself fail to account for these vast disparities in initial conditions; it is impossible to conceive of these disparities existing on the scale that they do without 'an organized state of civilization' [114] to uphold them.

3. There is evidence that economic institutions operating on an international scale (the 'organized state of civilization') have been complicit in upholding these injustices. Persons involved in upholding these institutions are thus implicated in creating subsequent ill health, even though they may be half-way around the world [115].

The moral implication is not only one of 'rectification' through strengthened human rights and more progressive systems of global resource redistribution; but also an obligation to change the way by which the rules of economic governance are established in order to overcome the historic and radical inequalities in initial conditions.

#### Global Health Equity Concerns

If moral concern with health-compromising global inequalities in resources and power begins with differences in peoples' initial conditions, the distinctions between equality of opportunity (procedural justice) and equality of outcome (substantive justice) lessen. Equality of opportunity, to be just, requires a disproportionate provision of public goods and capability resources for those whom history's conquests, and today's political institutions, place in highly unequal initial conditions. What becomes morally important is that all people have 'equal realization of their health potential' [[108], p. 431], a sentiment essentially similar to the 'right to highest attainable standard of physical and mental health.' What remains at issue is the extent of moral (or legal) obligation for amelioration of gross inequalities in initial conditions that create 'shortfall inequalities in central health capabilities' [[108], p. 431].

There is no answer to this question, apart from the imperative to seek one. In this quest, norms of procedural justice become important. Boggio [116], in an argument for why international organizations and those within them have an ethical obligation to act to redress systematic health inequalities, addresses *how *such policy decisions can be made in a just manner. He identifies three basic principles for an 'ethically-informed deliberative process': publicity (transparency in process, a comprehensible rationale, and public argument and evidence); relevance (trust in actors/institutions by recipients, opportunity for wide participation, and interventions based on recipients' needs, values and aspirations); and revisability (policies and programs can be challenged over time and improved, and individuals and institutions can be held accountable to purpose). Several of these conditions are similar to principles of good governance widely held by governments and multilateral organizations; that is, they can be considered as having a broad normative base. Citing Daniels [117], Boggio concludes that 'ethics require "that there is a space for deliberation in which reasonable people will disagree about what is ethically required, either from a human rights perspective or from other ethical conceptions"' [116]. These procedural elements of ethical decision-making, developed to apply to international institutions, could apply equally to negotiations encountered in global health diplomacy. These procedural elements, however, do not and should not reduce to communitarianism, in which the distributive norms and values of a community or society are taken as absolute regardless of their form, as long as there has been some deliberative space for sharing of differing conceptions of the 'good life' and 'justice' in their historic development. Such relativism would deny the powerful and evidence-informed justice arguments brought forward by Sen, Nussbaum and Pogge, amongst others.

## Conclusion

Global health is an increasingly prominent challenge to foreign policy deliberations. How should this challenge be framed with respect to improving global health equity? The assumption underlying our examination of differing global health frames is that each one sets the boundaries of problem-definition and intervention. In that sense, each frame examined has limitations but all have something strategic to offer (see Table 2).

**Table 2:** Summary of Key Arguments for Health in Foreign Policy

***Security ***gives global health interventions greater traction across a range of political classes than a rights-based argument alone. To the extent that this strengthens a base of public health expansion, securitisation of health may be a prerequisite to its eventual de-securitization. But vigilance is needed to avoid national security from trumping human security.	***Trade ***can improve health through global market integration, economic growth and positive health externalities. However, present trade rules skew benefits towards more economically and politically powerful countries; and evidence of negative health externalities demands careful *a priori *assessments of trade treaties for their health, development and human rights implications.
***Development ***remains the invitation to global governance debates. It provides a seat at the table. Risks inherent in its 'investing in health' instrumentalism can be tempered by continuously reminding decision makers to distinguish which one is the objective (human development) and which one the tool (economic growth).	***Human rights***, though weak in global enforcement, has advocacy traction and legal potential within national boundaries. Such rights do not resolve embedded tensions between the individual and the collective, an issue to which human rights experts are now attending

***Global public good****s *provides a language by which economists of one market persuasion can convince economists of another that there is a sound rationale for a system of shared global financing and regulation.	***Moral/ethical reasoning ***is suggested as a necessary addendum to the legalistic nature of human rights treaties. This need, in turn, has created scholarly momentum to articulate more rigorous argument for a global health ethic based on moral reasoning. Competitors for such an ethic range from a liberal theory of assistive duties based on 'burdened societies' in need, to cosmopolitan arguments that emphasise minimum capabilities needed for people to lead valued lives, to more recent arguments for a new ethic of relational justice based on cosmopolitan and human rights theories.

With respect to the normative goal of improving global health equity, a moral language is requisite for without an ethical reference point considerations other than national interests would likely prevail. But ethical argument in itself is insufficient as a basis for inserting health more forcefully into foreign policy-making. Legal language, setting forth the rules of national and global governance, is also needed and remains best provided in human rights covenants. Neither moral nor legal argument, in the absence of enforcement mechanisms, is necessarily compelling as an economic or political rationale. Economically, both the global public goods and development frames have some health utility in foreign policy debates, but only if they are located beneath a penumbra of ethical reasoning and legal obligation. Otherwise the risk exists that these discourses will lead to a triaging of foreign policy and global health aid decisions that reflect the interests of wealthier nations. Politically, the security and trade frames are the most potent but remain the most problematic. Both privilege existing relations of political, military and economic power over human rights, need or moral reasoning. The securitization of health, even in its human rather than national or economic rendering, remains premised in a conception of the individual made capable to function as a market actor; that is, it supports, rather than challenges, the social and economic assumptions that have driven the past three decades of neoliberal globalization [118].

While these differing policy frames offer multiple rationales for positioning health higher in foreign policy debates, what are the prospects that 'global health diplomats' will succeed in capturing more of the foreign policy turf? In partial answer, Fidler offers three conceptualizations to clarify global health's recent rise in foreign policy prominence: revolution, remediation and regression [119]. *Revolution *argues that health's increasing role in foreign policy is transformative of the health-foreign policy nexus. Health collapses the traditional distinction between high and low politics and provides new political space in which health is an overriding normative value and the ultimate goal of foreign policy. This conceptualization is consistent with health as a human right informed by moral/ethical reasoning. *Remediation *asserts that health's rise as a foreign policy issue reflects the continued reality of the traditional hierarchy of foreign policy functions. Health has become another issue that needs to be addressed through traditional approaches. It does not transform thinking and is not an overriding norm, and has risen as a foreign policy issue only because it threatens the high politics of national security and material interest. This conceptualization resonates most with health as security and, to some extent, health as development and as tradable commodity. *Regression *views health's integration into foreign policy as an indicator that health problems are getting worse. The increasing attention paid to health across the functions of foreign policy signifies the failure of public health efforts and a short-term need for some improvement to simply 'stay the course.'

Our desk review of existing policy and practice presented in this article largely concurs with the *remediation *conceptualization, consistent with the realist theory that states act in their own interests in the international arena. We are not alone in this assessment: others argue even more strongly that 'those global health issues for which a direct link to core economic, foreign and security interests is neither perceived nor proved will continue to be subjugated to other foreign policy priorities, regardless of the strength of the scientific evidence mustered in their favour' [[120], p.2]. This *realpolitik *conclusion has been argued for health and aid, trade, national security and even treaty-making, in which the revised IHRs and the FCTC as being 'driven by state interests which can either facilitate or undermine global health objectives' [[120], p.5]. National or mercantile self-interest, however, cannot entirely account for foreign policy practices. The push for increased maternal/child health aid, as we point out, reflects state interests only weakly and over a very long-term time horizon that is uncommon in the policy choices of short-mandate governments. Increased multilateral aid partly provided through global health initiatives further distances development assistance from donors' strategic interests. There is a disjunction between policy and practice in many instances, and inconsistencies within some of the policies themselves; but there is also argument offered that cautions against such lack of coherence. Coherence implies more than coordination across different policy sectors but, importantly, 'substantive consistency and synergy among different policies,' including 'consistency in framing how to analyze and address global health problems' [[121], p.14]. Our own analyses of coherence offered in this paper are suggestive only at this time; foreign policy is dynamic (as are changes in governments themselves), government actions are not driven solely by their policy pronouncements, and (importantly) policy statements or commentaries on health and foreign policy are still sufficiently new that *ex post *analyses of changes in government's foreign policy practices are unlikely to demonstrate any firm trend.

Although largely concurring that global health has yet to demonstrate any revolutionary shift in foreign policy drivers, to the extent that the different discursive framings for global health create an enlarged space for debate, an opening exists for global health equity to become more central in foreign policy deliberations. This challenges global health diplomats (whether government officials or representatives of civil society organizations) to strengthen the force of some of their arguments, notably with respect to trade (its economist limitations) but primarily in introducing human rights and ethical norms into foreign policy debate. Ethical arguments appear at present to be the most missing-in-action; and human rights arguments, with a few exceptions, make little or no reference to their legal standing or the international human rights framework (and accountability systems) of which they are a part.

There remains some cause for optimism that global health will retain and perhaps strengthen its prominence in foreign policy. Spain, during its EU presidency in the first half of 2010, focused on issues of global health equity, coherence and knowledge [122]. The WHO continues to emphasize the health risks of unregulated global financial markets while strengthening the knowledge and practice base for global health diplomacy. The transition from the G8 to the G20 (while still fraught with issues of economic elitism in global governance) incorporates some countries with stronger histories of rights-based approaches to health. And, despite the UN General Assembly in December 2009 focusing only on emerging infectious diseases and the critical shortage of health workers in many countries as health and foreign policy priorities [123], the background report to this resolution [124] was more comprehensive in scoping the global health foreign policy issues than were many of the government documents and commentaries examined in our own review.

Global health may thus be well-positioned to influence how globalization re-emerges from its present economic crisis; but how effectively it accomplishes this will partly be determined by the capacities and skills of its health diplomats, and the policy framing arguments they choose to emphasize or critique.

## Competing interests

The authors declare that they have no competing interests.

## Authors' contributions

RL conducted the initial research and wrote the first draft. MG reviewed the research, contributed additional material and commented on the first and subsequent drafts. Both authors read and approved the final manuscript.
